# Clinical characteristics and outcomes of invasive fungal infections in critically ill patients with liver cirrhosis

**DOI:** 10.1186/s12879-026-13709-5

**Published:** 2026-06-04

**Authors:** Jessica Seeßle, Elena Pelivan, Marietta Kirchner, Monica Boxberger, Patrick Michl, Uta Merle

**Affiliations:** 1 Department of Medicine IV, Gastroenterology, Hepatology and Infectious Diseases, University of Heidelberg, Heidelberg, Germany; 2 Institute of Medical Biometry, University of Heidelberg, Heidelberg, Germany

**Keywords:** IFI (invasive fungal infection), ICU (intensive care unit), Liver cirrhosis, ACLF (acute-on-chronic liver failure), Invasive candidiasis, Invasive pulmonary aspergillosis

## Abstract

**Background & aims:**

Invasive fungal infections (IFIs) are increasingly recognized as a severe and often underdiagnosed complication in critically ill patients with liver cirrhosis. Cirrhosis-associated immune dysfunction, and advanced disease stage contribute to an elevated risk, particularly in the intensive care unit (ICU). Despite their high mortality, data on the epidemiology and timing of IFIs in ICU-treated cirrhotic patients remain limited. We aimed to determine the prevalence, timing, and impact of IFIs on 30- and 90-day survival in this high-risk population.

**Methods:**

This retrospective single-center cohort study included 488 patients with liver cirrhosis who were admitted to ICU between January 2014 and December 2019. Patients were evaluated for the presence of IFIs during their hospital stay and stratified into Non-IFI (*n* = 458) and IFI (*n* = 30) groups. Comparative analyses were performed with a specific focus on invasive candidiasis and invasive pulmonary aspergillosis.

**Results:**

IFI prevalence was 6.1%. Invasive candidiasis and invasive pulmonary aspergillosis were diagnosed in 46.7% and 53.3% of IFI cases, with a median onset of 14.5 (6.50–24.00) and 17.5 (7.75-36.00) days, respectively. IFI patients had significantly more advanced liver disease reflected by higher MELD (*p* < 0.001) score and more severe ACLF (*p* = 0.009) and required significantly more invasive procedures such as dialysis (*p* < 0.001) parenteral nutrition (*p* < 0.001), and mechanical ventilation (*p* = 0.002). In addition, bacterial infections at admission were more common in IFI patients. The occurrence of IFI was associated with significantly reduced 30- and 90-day survival, particularly in patients with ACLF.

**Conclusion:**

These findings highlight the need for IFI surveillance in critically ill cirrhosis patients, particularly in those with ACLF and bacterial infections at admission.

## Introduction

Patients with liver cirrhosis are highly susceptible to infections, which can trigger liver decompensation and development of acute-on-chronic liver failure (ACLF) [1], resulting in a marked increase in mortality [1, 2]. While bacterial infections are well characterized in this population, invasive fungal infections (IFI) remain underrecognized despite their particularly poor prognosis. Reported mortality rates for IFIs, especially invasive candidiasis, approach 60% in patients with advanced liver disease [3–5].

The reported prevalence of IFIs in cirrhotic patients varies considerably depending on disease severity, diagnostic criteria, and patient population. A recent meta-analysis by Verma et al. demonstrated that 9.5% of cirrhotic patients develop proven or probable IFIs, with higher rates observed in critically ill patients and those with ACLF [6]. In contrast, substantially lower prevalence rates have been reported in ICU-specific cohorts and large multicentre studies, including the CANONIC study, where IFIs were observed in only 1–2% of patients [5, 7]. These discrepancies likely reflect differences in diagnostic intensity, study design, and patient selection.

Importantly, most IFIs in patients with cirrhosis are of nosocomial origin and tend to develop during hospitalization, particularly in the context of prolonged ICU stay, exposure to invasive procedures, and broad-spectrum antibiotic therapy [3, 5]. Identified risk factors include advanced liver disease, ACLF, renal dysfunction, prior bacterial infections, and ICU-related interventions such as mechanical ventilation and parenteral nutrition [3].

Despite increasing awareness, data on the epidemiology, timing, and clinical impact of IFIs in critically ill cirrhotic patients remain limited and inconsistent. Therefore, this study aimed to determine the prevalence, timing and impact of IFIs on 30- and 90-day survival of IFIs in a well-characterized cohort of cirrhotic patients admitted to the ICU.

## Materials and methods

### Study design

**Fig. 1:**
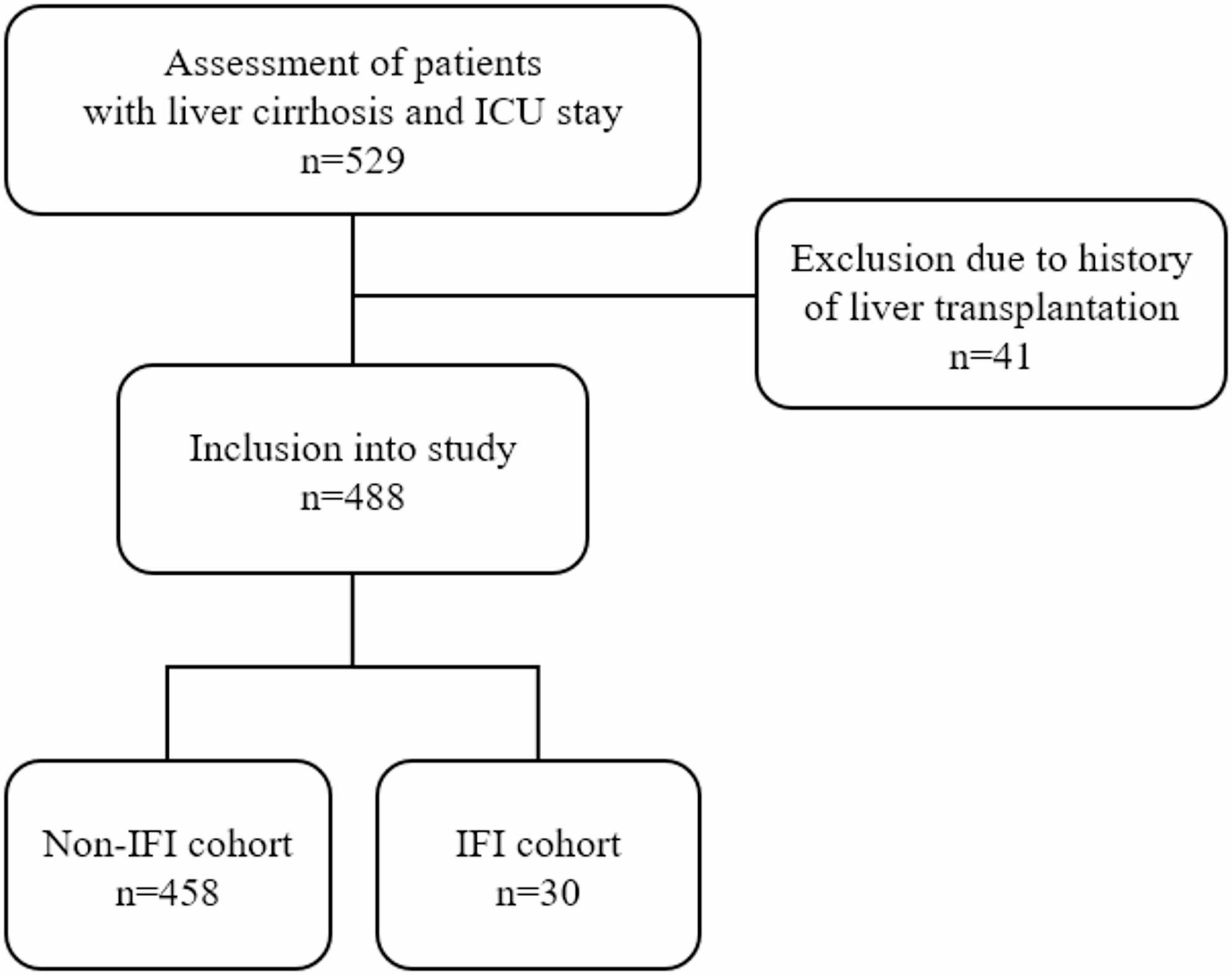
Flowchart of study inclusion with n = number of patients

This retrospective, single-center cohort study was conducted at the Department of Internal Medicine IV, University Hospital Heidelberg and covered the period from January 2014 to December 2019 (Ethics Committee of University of Heidelberg: reference number: S-457/2015). It included patients with confirmed liver cirrhosis who required intensive care treatment during hospitalization. The diagnosis was based on a combination of clinical and laboratory findings supported by imaging techniques (ultrasound, MRI, CT, elastography). In uncertain cases, histological analysis of liver biopsies was used for confirmation. Patients with a history of liver transplantation and subsequent cirrhosis of the transplanted liver were excluded due to the increased IFI risk from long-term immunosuppressive therapy. Similarly, patients who underwent liver transplantation during the study period were only included in the IFI group if the IFI diagnosis preceded the transplantation. A total of 529 patients were evaluated during the above-mentioned period, of whom 41 were excluded due to undergoing liver transplantation during their hospital stay. Finally, the study analyzed a total of 488 patients. Based on this assessment, two groups were defined: Non-IFI cohort (control cohort, *n* = 458) and IFI cohort (*n* = 30) (Fig. 1). All patients with invasive pulmonary aspergillosis included in our study fulfilled the criteria for probable or proven infection according to the revised consensus definitions of the European Organization for Research and Treatment of Cancer/Mycoses Study Group Education and Research Consortium (EORTC/MSGERC) [8]. Given that these criteria were originally developed for immunocompromised populations and are not fully validated in critically ill patients with liver cirrhosis, we adapted the definition of host factors by considering advanced liver cirrhosis as a relevant condition reflecting cirrhosis-associated immune dysfunction (CAID) [9], which is characterized by impaired innate and adaptive immune responses and increased susceptibility to infections. Probable IFI required compatible clinical and radiologic findings together with mycological evidence obtained from microbiological cultures, microscopy, or non-culture-based methods where available. Laboratory parameters were measured in the hospital central laboratory according to standard methods.

### Statistical analysis

The statistical analysis of this study is exploratory and thus, p-values are interpreted descriptively. The analysis was performed using IBM SPSS Statistics (Version 27). The significance level was set to 0.05. For skewed data, the median with interquartile range (IQR) and the Mann-Whitney U test were applied. Analyses of qualitative data were conducted using frequencies and Chi-square or Fisher’s exact test (in case of low counts in at least one category). Percentages were rounded to one decimal place and for all other values to two decimal places. The statistical analysis of time-to-event endpoints (overall survival) was based on the Kaplan-Meier method with the log-rank test to compare the estimated survival curves between groups. Survival curves are plotted with reporting of 30- and 90-days survival probabilities. In order to account for differences in severity of disease between IFI and Non-IFI cohort, a 1:1 matching for MELD score and ACLF grade was applied and outcomes concerning hospital and ICU stay were compared between the groups. Additionally, time to hospital discharge taking death as competing event was analyzed by Fine & Gray model in order to compare IFI and Non-IFI cohort adjusting for ACLF grade.

## Results

### Patient characteristics

The two cohorts were comparable in terms of demographic data including age, gender, etiology of liver cirrhosis and comorbidities. IFI patients had worse baseline liver function. Child-Pugh class A was absent in the IFI group (vs. 23.1% in Non-IFI), while class C was more frequent (56.7% vs. 29.3%). MELD score was significantly higher in the IFI cohort. ACLF was present at the time of hospital admission more often and with greater severity in IFI patients, reflected in higher CLIF-C OF scores. Details are summarized in Table 1.

**Table 1:** Baseline characteristics of the study cohort including demographic data, etiology of liver cirrhosis, relevant comorbidities, and disease severity scores at the time of hospital admission

	Non-IFI cohort (*n* = 458)	IFI cohort (*n* = 30)	*p*-value
Age (years)	58 (50.00–65.00)	58 (50.00–65.00)	0.220^b^
Gender (male)	307 (67.0%)	18 (60.0%)	0.429^a^
Etiology of liver cirrhosis			
Alcohol	273 (59.6%)	14 (46.7%)	0.402^a^
Autoimmune	30 (6.6%)	3 (10.0%)	
Viral (HBV or HCV)	46 (10.0%)	3 (10.0%)	
Cryptogenic	42 (9.2%)	3 (10.0%)	
MASLD	17 (3.7%)	0 (0.0%)	
Combined	27 (5.9%)	4 (13.3%)	
Viral (HBV + HDV or HCV)	3 (0.7%)	0 (0.0%)	
Viral + alcohol	17 (3.7%)	3 (10.0%)	
MASLD + alcohol	2 (0.4%)	0 (0.0%)	
Autoimmune + alcohol	1 (0.2%)	1 (3.3%)	
Autoimmune + MASLD	1 (0.2%)	0 (0.0%)	
Autoimmune + viral	1 (0.2%)	0 (0.0%)	
Budd-Chiari syndrome + alcohol	1 (0.2%)	0 (0.0%)	
Alpha-1 antitrypsin deficiency + drug-induced liver injury	1 (0.2%)	0 (0.0%)	
Others	23 (5.0%)	3 (10.0%)	
Wilson disease	3 (0.7%)	1 (3.3%)	
Hemochromatosis	3 (0.7%)	0 (0.0%)	
Drug induced liver injury	5 (1.1%)	0 (0.0%)	
Cardiac cirrhosis	5 (1.1%)	0 (0.0%)	
Budd-Chiari syndrome	1 (0.2%)	0 (0.0%)	
Secondary sclerosing cholangitis	1 (0.2%)	0 (0.0%)	
Connective tissue disease	1 (0.2%)	0 (0.0%)	
Progressive familial intrahepatic cholestasis	0 (0.0%)	1 (3.3%)	
Undetermined	4 (0.9%)	1 (3.3%)	
Pre-existing conditions			
Diabetes mellitus	126 (27.5%)	6 (20%)	0.370^a^
Chronic kidney disease	61 (13.3%)	5 (16.7%)	0.582^c^
Chronic dialysis	15 (3.3%)	3 (10.0%)	0.091^c^
Chronic lung disease	50 (10.9%)	4 (13.3%)	0.762^c^
Tumor disease	26 (5.7%)	2 (6.7%)	0.687^c^
Hepatocellular carcinoma	54 (11.8%)	5 (16.7%)	0.390^c^
Disease severity scores			
Child-Pugh score			
A	106 (23.1%)	0 (0.0%)	**< 0.001** ^**c**^
B	218 (47.6%)	13 (43.3%)	
C	134 (29.3%)	17 (56.7%)	
MELD score	21 (14.00–27.00)	27.50 (20.75–32.25)	**< 0.001** ^**b**^
ACLF grade			
0	212 (46.3%)	8 (26.7%)	**0.009** ^**c**^
1	100 (21.8%)	6 (20.0%)	
2	90 (19.7%)	8 (26.7%)	
3	56 (12.2%)	8 (26.7%)	
CLIF-C OF score	10 (9.00–11.00)	11 (9.75-14.00)	**0.020** ^**b**^
CLIF-C ACLF score	51 (45.00–58.00)	52.50 (49.50-62.75)	0.085^b^

Results are presented as median (IQR) or n (%). P-value is based on Chi-squared test (a), Mann-Whitney U test (b), or Fisher’s exact test (c). ACLF = acute-on-chronic liver failure, CLIF = chronic liver failure. HBV = hepatitis B virus, HCV = hepatitis C virus, IFI = invasive fungal infection, MASLD = metabolic dysfunction-associated steatotic liver disease, MELD = model of end-stage liver disease

### Laboratory findings

Table 2 provides a comparative overview of laboratory results at the time of hospital admission from both cohorts. Liver function markers were abnormal in both groups. INR was significantly higher in IFI patients (1.75 vs. 1.52, *p* = 0.001), while albumin was comparably reduced. Creatinine levels were elevated in both groups at admission, but significantly higher in the IFI group (1.9 mg/dL vs. 1.37 mg/dL, *p* = 0.020), indicating greater renal impairment. Inflammatory markers (CRP, PCT) were increased in both groups, with significantly higher levels in IFI patients. In summary, IFI patients presented with significantly worse liver function, higher inflammatory markers, and greater renal dysfunction at admission.

**Table 2:** Laboratory findings of the Non-IFI and IFI cohort at the time of hospital admission

	Non-IFI cohort (*n* = 458)	IFI cohort (*n* = 30)	*p*-value
Aspartate Aminotransferase, U/l	82 (44.00-152.25)	108 (63.00-241.00)	0.078
Alanine Aminotransferase, U/l	35 (21.00-66.25)	62 (32.50-105.25)	**0.026**
Bilirubin, mg/dl	3.01 (1.30–8.70)	6.10 (1.45–15.90)	**0.039**
Creatinine, mg/dl	1.37 (0.79–2.42)	1.90 (1.06–3.87)	**0.020**
International normalized ratio	1.52 (1.27–1.86)	1.75 (1.44–2.30)	**0.001**
Albumin, g/l	27.10 (23.20–31.30)	28.65 (23.40-29.95)	0.593
Hemoglobin, g/dl	9.50 (7.90–11.50)	8.95 (7.88–10.43)	0.230
White cell count, /µl	9.46 (6.28–14.36)	12.38 (5.80-15.65)	0.175
Platelets, /nl	106 (58.00-169.25)	80.50 (58.75–152.50)	0.609
C reactive protein, mg/l	24.20 (10.48–55.73)	42.85 (22.53-132.13)	**0.004**
Procalcitonin, µg/l	0.51 (0.20–1.56)	1.12 (0.58–2.95)	**0.006**

Results are presented as median (IQR) and p-value is based on Mann-Whitney U test. IFI = invasive fungal infection

### Hepatic complications, interventions, and outcomes in IFI and Non-IFI patients

IFI patients had markedly worse outcomes compared to Non-IFI patients. Median hospital and ICU stays were significantly longer (hospital: 33 vs. 13.5 days, *p* < 0.001; ICU: 20 vs. 4.5 days, *p* < 0.001), and ICU survival was significantly lower (36.7% vs. 73.4%, *p* < 0.001) in IFI patients. Only 33.3% of IFI patients were discharged from hospital, compared to 72.9% in the Non-IFI cohort (*p* < 0.001). Ascites was present in all IFI patients vs. in 66.6% in Non-IFI (*p* = 0.001). Hepatic encephalopathy was more frequent and severe in IFI patients (HE > 2: 20% vs. 13.3%), though this difference did not reach statistical significance (*p* = 0.065). Non-variceal bleeding occurred significantly more often during hospitalization in IFI patients (33.3% vs. 14.6%, *p* = 0.016) and acute kidney injury was also more prevalent (80% vs. 47.2%, *p* < 0.001). Invasive procedures prior to IFI diagnosis were significantly more common in the IFI cohort compared to the Non-IFI cohort. Dialysis was required in 56.7% of IFI patients versus 23.4% in the Non-IFI cohort (*p* < 0.001). Parenteral nutrition (76.7% vs. 34.9%, *p* < 0.001) and invasive mechanical ventilation (66.7% vs. 37.3%, *p* = 0.002) were also more frequently applied in the IFI group. Regarding medication use, 86.7% of IFI patients received antibiotics for more than five days, compared to 63.1% in the Non-IFI cohort (*p* = 0.009). A summary can be found in Table 3.

**Table 3:** Hepatic complications, interventions, and outcomes of the Non-IFI and IFI cohort on admission and during hospital stay

	Non-IFI cohort (*n* = 458)	IFI cohort (*n* = 30)	*p*-value
Hepatic decompensation			
Ascites	305 (66.6%)	30 (100.0%)	**< 0.001** ^**a**^
Hepatic encephalopathy			0.065^c^
none	301 (65.7%)	15 (50.0%)	
0 < HE ≤ 2	96 (21.0%)	9 (30.0%)	
HE > 2	61 (13.3%)	6 (20.0%)	
Acute kidney injury	216 (47.2%)	24 (80.0%)	**< 0.001** ^**a**^
Bleeding complications			
Variceal bleeding at admission	84 (18.3%)	3 (10%)	0.328^c^
Variceal bleeding during hospital stay	25 (5.5%)	2 (6.7%)	0.678^c^
Non-variceal bleeding at admission	93 (20.3%)	8 (26.7%)	0.484^c^
Non-variceal bleeding during hospital stay	67 (14.6%)	10 (33.3%)	**0.016** ^**c**^
Invasive procedures			
Dialysis	107 (23.4%)	17 (56.7%)	**< 0.001** ^**c**^
Surgical procedure	54 (11.8%)	7 (23.3%)	0.082^c^
Parenteral nutrition	160 (34.9%)	23 (76.7%)	**< 0.001** ^**a**^
Mechanical ventilation	171 (37.3%)	20 (66.7%)	**0.002** ^**a**^
Steroids	45 (9.8%)	5 (16.7%)	0.182^c^
Antibiotics > 5 days	289 (63.1%)	26 (86.7%)	**0.009** ^**a**^
Duration of ICU stay (days)	4.5 (2.00–11.00)	20 (10.25–80.25)	**< 0**.**001**^**b**^
Duration of hospital stay (days)	13.5 (6.00-27.25)	33 (15.75-104.25)	**< 0.001** ^**b**^
Survived ICU stay	336 (73.4%)	11 (36.7%)	**< 0.001** ^**a**^
Survived hospital stay	334 (72.9%)	10 (33.3%)	**< 0.001** ^**a**^

Results are presented as median (IQR) or n (%). P-value is based on Chi-squared test (a), Mann-Whitney U test (b), or Fisher’s exact test (c). ICU = intensive care unit, IFI = invasive fungal infection, IQR = interquartile range

**Table 4:** Results are presented as median (IQR) or n (%)

	Matched Non-IFI cohort (*n* = 30)	IFI cohort (*n* = 30)	*p*-value
MELD score	27.50 (20.50-32.25)	27.50 (20.75–32.25)	0.778^b^
ACLF grade			
0	8 (26.7%)	8 (26.7%)	1.00^a^
1	6 (20.0%)	6 (20.0%)	
2	8 (26.7%)	8 (26.7%)	
3	8 (26.7%)	8 (26.7%)	
Duration of ICU stay (days)	4.00 (2.00-15.50)	20.00 (10.25–80.25)	**< 0.001** ^**b**^
Duration of hospital stay (days)	19.5 (6.00–36.50)	33.00 (15.75-104.25)	**0.010** ^**b**^
Survived ICU stay	19 (63.3%)	11 (36.7%)	**0.039** ^**a**^
Survived hospital stay	19 (63.3%)	10 (33.3%)	**0.020** ^**a**^

P-value is based on Chi-squared test (a), Mann-Whitney U test (b), ACLF = acute-on-chronic liver failure, IFI = invasive fungal infection, IQR = interquartile range, MELD = model of end-stage liver disease

To assess whether the severity of liver disease influenced the in-hospital course and outcomes, patients in the IFI cohort were matched to patients in the non-IFI cohort according to MELD score and ACLF grade. After matching, both groups showed comparable disease severity, with no significant differences in MELD score (27.5 vs. 27.5, *p* = 0.778) or ACLF grade (*p* = 1.00, Table 4)). Despite the comparable baseline severity of liver disease, significant differences in clinical outcomes remained between the two groups. Patients in the IFI cohort had a significantly longer ICU stay compared to the matched non-IFI cohort (20.0 vs. 4.0 days, *p* < 0.001). Similarly, the duration of hospital stay was significantly prolonged in IFI patients (33.0 vs. 19.5 days, *p* = 0.010). Furthermore, survival outcomes differed significantly between the groups. Patients with IFI were significantly less likely to survive the ICU stay (36.7% vs. 63.3%, *p* = 0.039) and the overall hospital stay (33.3% vs. 63.3%, *p* = 0.020). Overall, these findings suggest that the presence of IFI was associated with prolonged hospitalization and poorer survival independent of the underlying severity of liver disease as reflected by MELD score and ACLF grade at admission.

**Table 5:** Competing risk analysis for time to discharge from hospital with death as competing event unadjusted (only cohort factor included) and adjusted for ACLF grade

	Parameter estimate	Standard error	Hazard ratio	95% Hazard Ratio Confidence Limits		*p*-value
IFI vs. Non-IFI	-1.279	0.287	0.278	0.159	0.488	**< 0.0001**
Adjusted for ACLF grade						
IFI vs. Non-IFI	-1.171	0.275	0.310	0.181	0.531	**< 0.0001**
ACLF grade	-0.452	0.057	0.636	0.569	0.711	**< 0.0001**

ACLF = acute-on-chronic liver failure, IFI = invasive fungal infection

In the competing risk analysis for time to discharge with death as competing event, IFI was significantly associated with lower probability of hospital discharge (HR 0.278, 95% CI 0.159–0.488, *p* < 0.0001). After adjustment for ACLF grade, IFI remained independently associated with lower discharge probability (adjusted HR 0.310, 95% CI 0.181–0.531, *p* < 0.0001). In addition, higher ACLF grade was independently associated with lower probability of discharge (HR 0.636, 95% CI 0.569–0.711, *p* < 0.0001, Table 5).

### Incidence and clinical presentation of IFI cohort

Invasive candidiasis was diagnosed in 46.7% of patients (*n* = 14), with a median onset of 14.5 days after hospital admission (IQR: 6.5–24.0). Invasive candidiasis was categorized into candidemia with or without an identifiable focus and candida peritonitis without concomitant candidemia (Table 6). Candidemia was the predominant presentation (71.4%). In cases of candidemia, identified sources of the infection included Candida peritonitis (30%), urogenital tract infections (20%) and catheter-associated infections (20%), while the source remained unknown in 20% of cases. Candida peritonitis without candidemia was observed in four patients (28.6%).

Invasive pulmonary aspergillosis was diagnosed in 53.3% of patients (*n* = 16) within the IFI cohort, with a median onset of 17.5 days after hospital admission (IQR: 7.75–36.00). All patients met the criteria for proven or probable infection, as described in Methods section. In our cohort, microbiological confirmation of Aspergillus fumigatus from bronchoalveolar lavage (BAL) was achieved in 4 out of 16 patients. In two of these patients, the first BAL sample obtained was already positive for Aspergillus fumigatus. In another patient, a tracheal secretion collected two days prior to BAL sampling was negative, whereas the subsequent BAL demonstrated significant growth of Aspergillus fumigatus (> 10^5 CFU). The fourth patient underwent three sequential BAL procedures at intervals of two days, and only the third BAL sample yielded a positive result for Aspergillus fumigatus. None of the IFI patients were receiving antifungal therapy at the time of diagnosis. The therapy was initiated upon diagnosis.

Taken together, these findings underscore that repeated microbiological sampling is often necessary, particularly in critically ill ICU patients, initial respiratory samples may remain negative despite ongoing infection. Therefore, a high level of clinical suspicion and repeated diagnostic evaluation, including sequential BAL sampling when appropriate, are essential, as the diagnosis of invasive pulmonary aspergillosis can be challenging and delayed.

**Table 6:** Invasive candidiasis and invasive pulmonary aspergillosis of the IFI cohort

	IFI cohort (*n* = 30)
**Invasive Candidiasis**	**14 (46.7%)**
Onset (days after admission)	14.50 (6.50–24.00)
Candidemia	10 (71.4%)
Focus of candidemia:	
Peritonitis	3 (30.0%)
Urogenital	3 (30.0%)
Catheter-associated	2 (20.0%)
Unknown focus	2 (20.0%)
Candida peritonitis without candidemia	4 (28.6%)
**Invasive pulmonary aspergillosis**	**16 (53.3%)**
Onset (days after admission)	17.5 (7.75–36.00)

Results are presented as median (IQR) or n (%). IFI = invasive fungal infection, IQR = interquartile range

### Summary of bacterial infections at admission and time to IFI diagnosis in the IFI cohort

**Fig. 2:**
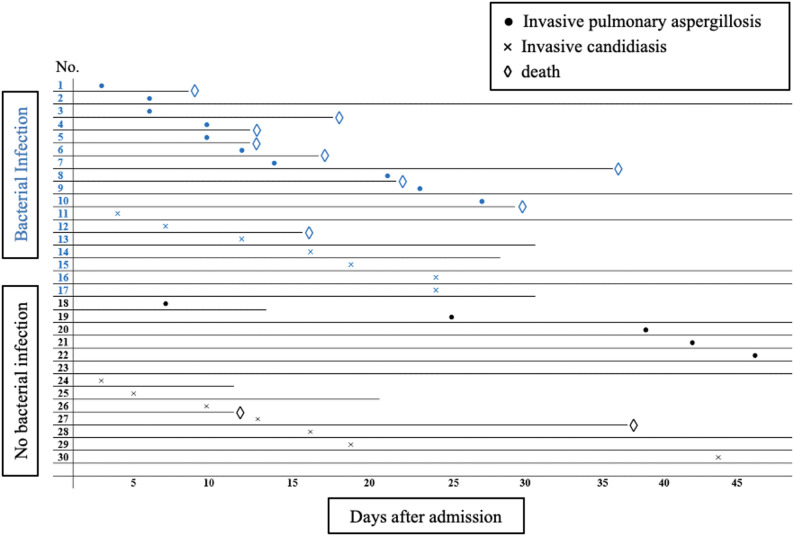
IFI cohort (*n* = 30) with bacterial infections at time of admission and time to IFI diagnosis (days). A time line of 50 days is shown. The blue marking corresponds to patients who had a bacterial infection at the time of admission. Events are indicated: dot = invasive pulmonary aspergillosis, cross = invasive candidiasis, rhombus = death. IFI = invasive fungal infection

Among the 30 patients in the IFI cohort, 17 (56.7%) had a bacterial infection at the time of hospital admission, while 13 (43.3%) showed no evidence of bacterial infection. The most common infection was spontaneous bacterial peritonitis, observed in 10 patients, followed by cholangitis (*n* = 1), secondary peritonitis (*n* = 1), pleural empyema (*n* = 1), community-acquired pneumonia (CAP, *n* = 1), urosepsis (*n* = 1), and infections of unknown focus (*n* = 2) (Fig. 2). The median time to IFI diagnosis (including invasive candidiasis and invasive pulmonary aspergillosis) after admission varied, ranging from 3 to 100 days. For patients with invasive pulmonary aspergillosis, time to diagnosis ranged from 3 to 46 days, while for those with invasive candidiasis, it ranged from 3 to 44 days.

**Fig. 3:**
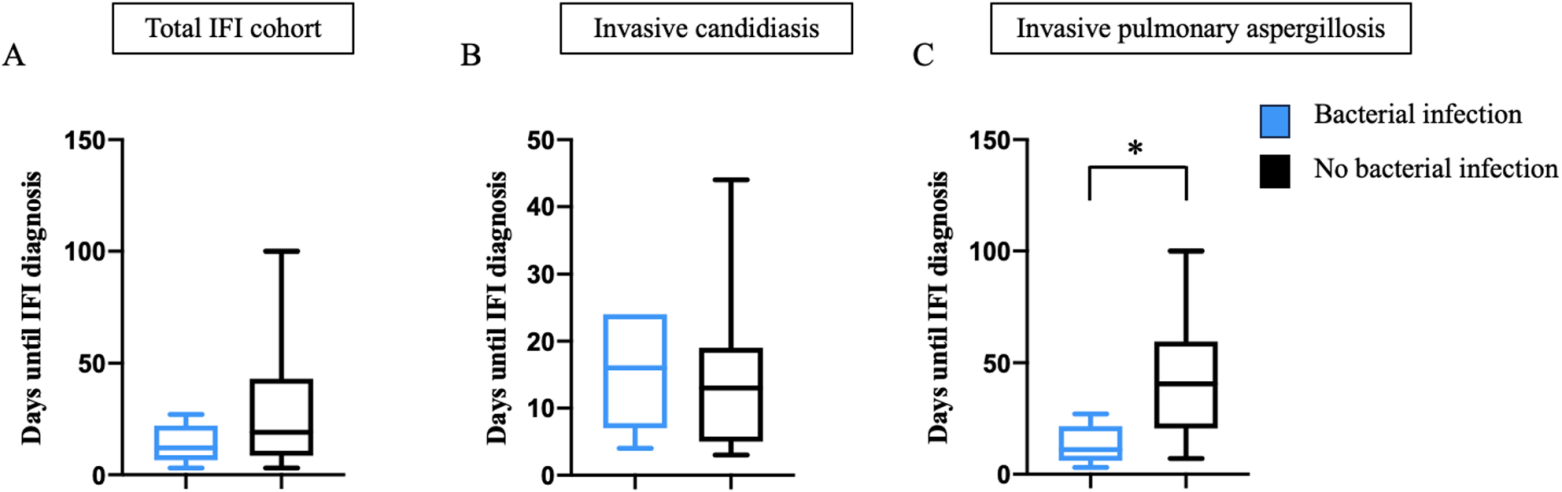
Bacterial infection at hospital admission and the time from admission to diagnosis of invasive fungal infection (IFI). (**A**) total IFI cohort (*n* = 30) (**B**) subgroup of invasive candidiasis (*n* = 14) and (**C**) invasive pulmonary aspergillosis (*n* = 16), * *p* = 0.05

Patients with bacterial infections tended to receive an IFI diagnosis earlier than those without bacterial infections at admission (Fig. 3), although this difference was not statistically significant in the overall IFI cohort. In contrast, within the subgroup of invasive pulmonary aspergillosis, patients with bacterial infections were diagnosed with IFI significantly earlier than those without bacterial infections (*p* = 0.016). This finding suggests a possible association between prior bacterial infection and an earlier IFI onset in this cohort. Overall, the high prevalence of bacterial infections at baseline highlights the complexity of managing co-infections in critically ill patients.

### Overall survival in the IFI cohort

**Fig. 4:**
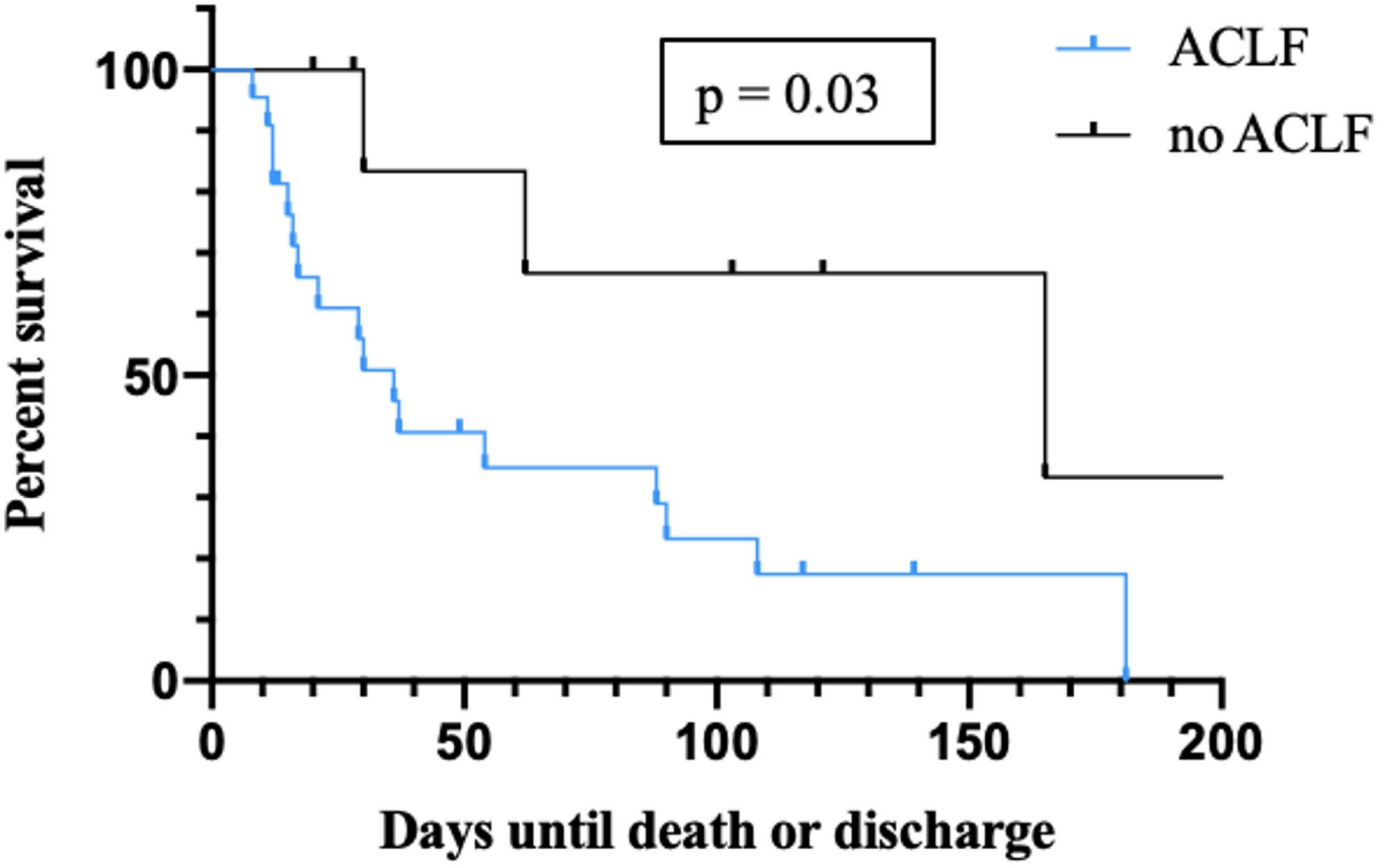
Kaplan-Meier curve showing survival probabilities of the IFI cohort during hospital stay by the presence or absence of ACLF at admission. ACLF = acute-on-chronic liver failure, IFI = invasive fungal infection

For the IFI cohort, the in-hospital 30-day survival rate was 59.9%. The 90-day survival was 34.6%. To better characterize survival within the IFI cohort and identify potential prognostic factors, survival probabilities were compared between IFI patients with and without ACLF at the time of admission. The 30-day survival probability was 83.3% in patients without ACLF at admission, compared to 50.8% in those with ACLF. At 90 days, survival was 66.7% without ACLF and 23.2% with ACLF. The differences in survival were statistically significant (*p* = 0.03, Fig. 4).

**Fig. 5:**
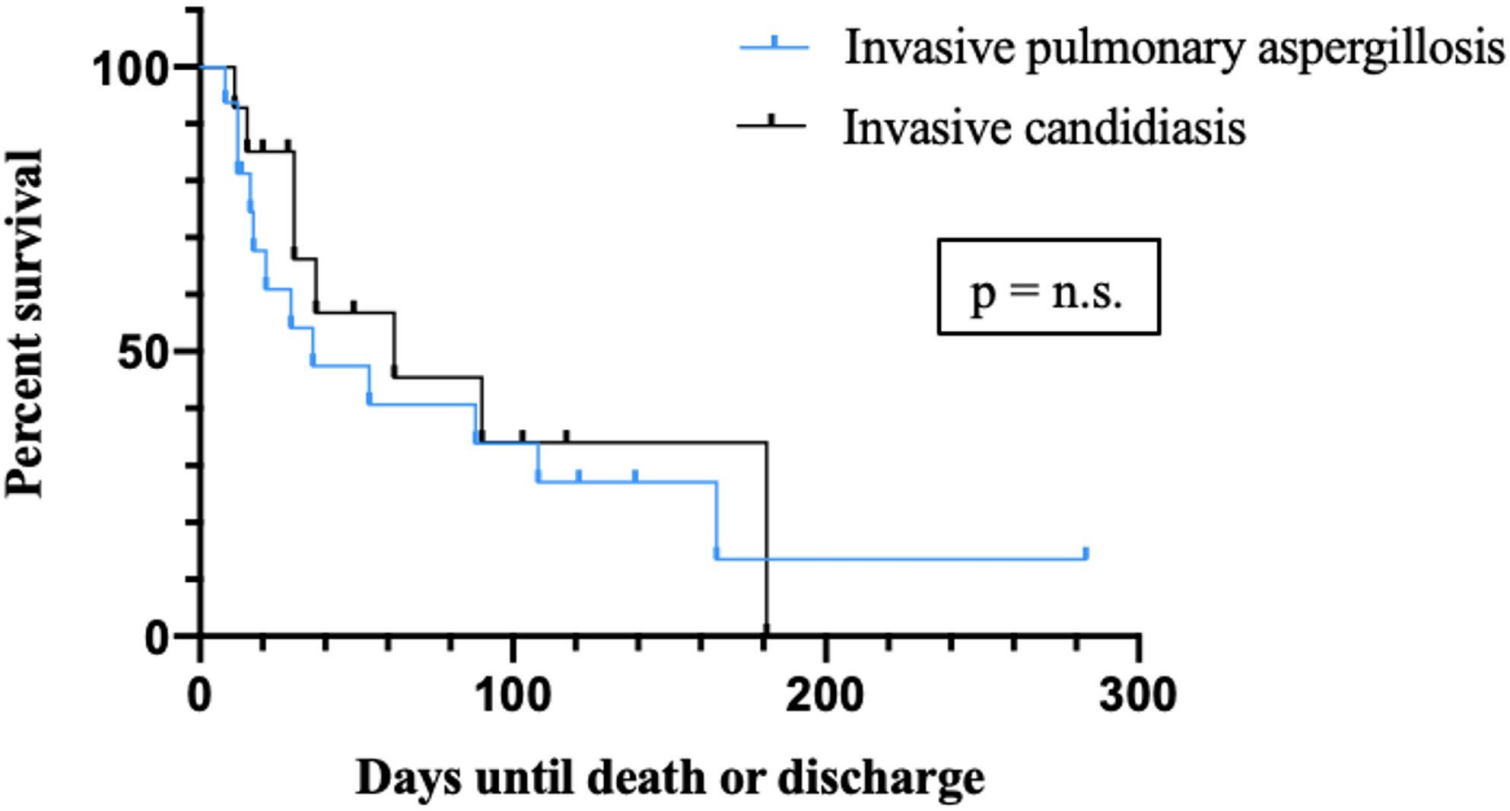
Kaplan-Meier curve showing survival probabilities of the IFI group during hospital stay stratified by invasive pulmonary aspergillosis and invasive candidiasis. IFI = invasive fungal infection

In contrast, the pathogen species causing the IFI appeared to have less impact on patient survival. Thirty days after diagnosis, the survival rate was 54.2% for patients with invasive pulmonary aspergillosis and 66.2% for those with invasive candida infections. At 90 days, survival among hospitalized patients was 33.% for those with invasive pulmonary aspergillosis and 34.1% for those with invasive candidiasis. No statistically significant difference was found between the two survival curves (Fig. 5).

## Discussion

Invasive fungal infections (IFIs) represent a significant but underrecognized complication among critically ill patients with liver disease, particularly those admitted to intensive care units. Unlike many prior studies that included general hospital cohorts or mixed cases of colonization and infection, our study strictly focused on ICU patients and included patients with invasive candidiasis with and without candidemia and invasive pulmonary aspergillosis.

Our findings suggest that advanced liver disease at ICU admission is associated with an increased risk of IFIs. This observation is consistent with previous reports demonstrating a higher prevalence of fungal infections among patients with more severe hepatic dysfunction [3, 4, 6, 10]. Together, these data support the concept that critically ill patients with advanced liver disease and ACLF are particularly vulnerable to opportunistic infections, including IFIs. This increased susceptibility is likely multifactorial and may be driven by cirrhosis-associated immune dysfunction (CAID), which is characterized by impaired innate and adaptive immune responses in the setting of persistent systemic inflammation [9], as well as by the frequent need for invasive procedures and intense organ support.

Patients with IFI showed significantly worse clinical outcomes, including prolonged ICU stay, higher ICU mortality, and reduced overall survival. Outcomes were particularly poor among patients with concomitant ACLF, in line with previous reports demonstrating high mortality rates in patients with IFI and advanced liver failure [10, 11]. To account for these differences in disease severity, matched analyses incorporating MELD and ACLF, as well as competing risk analysis adjusted for ACLF, were performed. Even after adjustment, IFI remained associated with poorer overall-, hospital-, and ICU-survival, suggesting that IFI may represent an independent contributor to adverse outcomes. Nevertheless, as patients with IFI were generally more critically ill, suggesting that IFIs may, at least in part, represent a marker of advanced disease severity and ongoing clinical deterioration rather than an independent driver of poor outcome. Accordingly, residual confounding cannot be excluded, and the extent to which IFIs causally contribute to adverse outcomes warrants further investigation.

In our cohort, the median time to IFI diagnosis was 15 days after ICU admission, indicating that fungal infections frequently developed during the later course of critical illness rather than being present at admission as community-acquired infections. This temporal pattern may support the hypothesis that IFIs in critically ill patients with liver disease are predominantly nosocomial complications, associated with prolonged ICU treatment, repeated invasive interventions, and extended exposure to broad-spectrum antimicrobial therapy [5, 6]. The comparatively late occurrence of IFI highlights the importance of maintaining clinical awareness throughout the ICU stay, particularly in patients with prolonged hospitalization or unexplained clinical deterioration. Because IFI symptoms are often non-specific and may overlap with bacterial infections or progression of liver failure, delayed diagnosis remains challenging and may postpone appropriate antifungal treatment.

The identification of factors associated with IFIs may facilitate earlier recognition of patients at increased risk and thereby supporting timely diagnostic and therapeutic strategies. In our cohort, ascites and acute kidney injury at ICU admission were associated with an increased risk of subsequent IFI development. In addition, IFI patients presented more frequently with bacterial infections at admission, a finding that was significant in the subgroup of invasive pulmonary aspergillosis. These associations may reflect possibly advanced liver disease and impaired host defense mechanisms. Furthermore, several in-hospital factors, like invasive procedures (hemodialysis, surgery, mechanical ventilation), parenteral nutrition, and prolonged antibiotic therapy (> 5 days) were more frequently observed in patients with IFI. These findings align with previous studies that IFI development in critically ill patients with advanced liver disease is multifactorial and likely influenced by both underlying disease severity and ICU-related exposure [3, 7].

Due to the limited sensitivity and specificity of current diagnostic tests, the diagnosis of invasive IFIs typically relies on different levels of diagnostic certainty, as defined by the international EORTC/MSG Consensus Group, namely proven, probable, and possible IFIs [8]. However, these criteria were originally developed for immunocompromised patients and have limited applicability to critically ill ICU population, where their diagnostic performance is less reliable [8]. Therefore, the criteria for invasive pulmonary aspergillosis and invasive candidiasis have been challenged in ICU settings, leading to the development of alternative diagnostic algorithms [12–14]. To address these limitations, the FUNDICU consensus project was established to develop standardized definitions of specifically for critical ill ICU patients with the aim to improve research comparability and clinical management in this high-risk population [15]. Recent findings underscore the importance of adapting diagnostic criteria to ICU-specific conditions and comorbidities, including decompensated liver cirrhosis. For the first time, FUNDICU provides clear recommendations for the classification of probable IFIs in ICU patients [16].

Diagnostic challenges are particularly pronounced in patients with liver disease. In this population, conventional fungal biomarkers such as 1,3-β-D-glucan and galactomannan may have limited diagnostic accuracy because false-positives occur due to bacterial infections, medical interventions, or fungal colonization. At the same time, the low sensitivity of current diagnostic tests and the often non-specific clinical presentation of IFIs contribute to delayed or missed diagnosis. The use of the adapted EORTC/MSG criteria in our cohort represents a methodological strength by providing a standardized framework for IFI classification. Nevertheless, these criteria may still underestimate probable or possible IFIs in patients with liver disease. Therefore, biomarkers such as β-D-glucan and galactomannan should be interpreted as complementary diagnostic tools rather than definitive markers [6].

The distinction between fungal colonization and true invasive infection remains one of the major challenge in clinical practice, particularly in critical ill and immunocompromised patients. Although this study focused on proven and probable invasive infections, the transition from simple colonization to tissue-invasive disease is often difficult to define in routine clinical settings. Therefore, future research should focus on the development of more sensitive and specific diagnostic tools to better distinguish colonization from true invasive infection. More accurate differentiation would support earlier and more targeted antifungal therapy while minimizing overtreatment, toxicity, and the emergence of antifungal resistance.

The significantly worse outcomes observed in IFI patients underscore the need for early identification and aggressive management. However, the optimal approach to antifungal therapy in critically ill liver disease patients remains debated. While early empirical treatment may improve outcomes in high-risk individuals, indiscriminate use of antifungals can lead to drug toxicity, adverse drug interactions, and the emergence of resistant strains. Balancing these considerations requires a nuanced approach to risk stratification and treatment decision-making. While current guidelines suggest considering antifungals in patients with risk factors (e.g., prolonged antibiotics, central venous access, ACLF), the decision must balance timely treatment against antifungal overuse [17].

In our study, no significant difference in mortality was observed between patients with invasive candidiasis and those with invasive pulmonary aspergillosis. This finding is noteworthy, as previous literature, has generally reported higher mortality in invasive pulmonary aspergillosis compared with candidemia, particularly in critically ill patients with liver cirrhosis [18]. This finding may be related to the limited sample size and should therefore be interpreted with caution. Consequently, it remains unclear whether the absence of a mortality difference reflects a true clinical effect or is attributable to limited statistical power. Further studies in larger, preferably multicenter cohorts are warranted to validate these findings and to better characterize potential outcome differences between invasive pulmonary aspergillosis and candidemia.

This study has several limitations that should be considered when interpreting the findings. The retrospective single-center design may limit the generalizability of the results and introduces the potential for selection and information bias. Residual confounding, especially by disease severity (e.g., MELD score and ACLF), cannot be fully excluded and may have influenced the observed associations. Furthermore, the relatively limited number of IFI cases restricted the statistical power for subgroup analyses and may have limited the identification of additional independent risk factors. Finally, due to the observational nature of the study, causal relationships between identified risk factors and IFI development cannot be definitively established of treatment adequacy or resistance patterns.

In conclusion, invasive fungal infections represent a serious and underrecognized complication in ICU patients with liver disease, particularly those with ACLF, prolonged ICU stay, invasive procedures, and extensive antibiotic exposure. Our findings support enhanced fungal surveillance and a risk-adapted management approach in high-risk patients. In addition to timely antifungal therapy, prevention strategies such as antibiotic stewardship, reduction of unnecessary invasive procedures, and the integration of novel biomarkers and molecular diagnostics may improve early detection and clinical outcomes. Overall, our findings support the growing recognition that IFI should be considered not only as a marker of disease severity but also as a modifiable determinant of outcome in this vulnerable population. Future research should focus on developing and validating risk prediction models tailored to critically ill patients with liver disease incorporating both baseline liver disease severity and in-hospital risk factors.

## Data Availability

The datasets used and analyzed during the current study are available from the corresponding author on reasonable request.

## Abbreviations

ACLF: Acute-on-chronic liver failure
AKI: Acute kidney injury
BAL: Bronchoalveolar lavage
CAID: Cirrhosis-associated immune dysfunction
CAP: Community-acquired pneumonia
CFU: Colony forming units
CKD: Chronic kidney disease
CLIF: Chronic liver failure
CRP: C reactive protein
HBV: Hepatitis b virus
HCC: Hepatocellular carcinoma
HCV: Hepatitis c virus
HE: Hepatic encephalopathy
ICU: Intensive care unit
IFI: Invasive fungal infection
IQR: Interquartile range
MASLD: Metabolic associated steatotic liver disease
MELD: Model of end-stage liver disease
PCT: Procalcitonin
SBP: Spontaneous bacterial peritonitis
